# Identification of a rickettsial endosymbiont in a soft tick *Ornithodoros turicata americanus*

**DOI:** 10.1371/journal.pone.0278582

**Published:** 2022-12-06

**Authors:** Lichao Liu, Daniel E. Sonenshine, Hameeda Sultana, Girish Neelakanta

**Affiliations:** 1 Department of Biomedical and Diagnostic Sciences, College of Veterinary Medicine, University of Tennessee, Knoxville, TN, United States of America; 2 Department of Biological Sciences, Old Dominion University, Norfolk, VA, United States of America; 3 Vector Molecular Biology Section, Laboratory of Malaria and Vector Research, National Institute of Allergy and Infectious Diseases, National Institutes of Health, Rockville, MD, United States of America; University of Kentucky College of Medicine, UNITED STATES

## Abstract

Bacterial endosymbionts are abundantly found in both hard and soft ticks. *Occidentia massiliensis*, a rickettsial endosymbiont, was first identified in the soft tick *Ornithodoros sonrai* collected from Senegal and later was identified in a hard tick *Africaniella transversale*. In this study, we noted the presence of *Occidentia* species, designated as *Occidentia*-like species, in a soft tick *O*. *turicata americanus*. Sequencing and phylogenetic analyses of the two genetic markers, 16S rRNA and *groEL* confirmed the presence of *Occidentia*-like species in *O*. *turicata americanus* ticks. The *Occidentia*-like species was noted to be present in all developmental stages of *O*. *turicata americanus* and in different tick tissues including ovaries, synganglion, guts and salivary gland. The levels of *Occidentia*-like species 16S rRNA transcripts were noted to be significantly higher in ovaries than in a gut tissue. In addition, *Occidentia*-like species *groEL* expression was noted to be significantly higher in tick synganglion than in ovaries and gut tissues. Furthermore, levels of *Occidentia*-like species 16S rRNA transcripts increased significantly upon *O*. *turicata americanus* blood feeding. Taken together, our study not only shows that *Occidentia*-like species is present in *O*. *turicata americanus* but also suggests that this bacterium may play a role in tick-bacteria interactions.

## Introduction

In the United States, soft ticks *Ornithodoros turicata* and *Ornithodoros hermsi* are the primary vectors for *Borrelia turicatae* and *Borrelia hermsii*, respectively, the causative agents of tick-borne relapsing fever (TBRF) in humans [1–4]. Recent reports suggest that *O*. *turicata* was also noted to be a potential vector that could transmit African swine fever virus (ASFV) to pigs (*Sus scrofa*) [5, 6]. These ticks are opportunistic, nidicolous feeders that feed rapidly and take a complete blood meal within 60 minutes [7–10]. Some of the recognized hosts for *O*. *turicata* ticks includes gopher tortoise, squirrels, snakes, cattle, pigs, and prairie dogs [7–10]. The developmental stages in the life cycle of *O*. *turicata* includes eggs, larvae, nymphs, and adults [7–10]. After mating with a male, female *O*. *turicata* ticks lay eggs that hatch into 6-legged larvae which feed on a small vertebrate host and molt into first-instar 8-legged nymphs [7–10]. Unlike hard ticks, *O*. *turicata* female ticks can mate and lay hundreds of eggs several times [7–11]. After a blood meal, the first-instar nymphs molt into second-instar stage nymphs [7–10]. *Ornithodoros turicata* has up to seven nymphal stages [7–10]. Ticks continue through multiple instar stages until molting into the adult stage where sexual differentiation occurs [7–10]. Laboratory studies have indicated that these ticks can survive extreme periods of starvation between blood meals and adult ticks can survive up to 10 years with a regular blood feeding [11].

In addition to harboring several human pathogens, both hard and soft ticks harbor many rickettsial bacteria, most of which are noted to be non-pathogenic [12–19]. *Rickettsia* is a genus comprised of diverse obligate intracellular gram-negative bacteria that are primarily transmitted by various arthropods, including ticks [20]. *Rickettsia* spp. are reported to be the most common endosymbionts in *Ixodes*, *Amblyomma*, and *Dermacentor* ticks, although less prevalent in the genera of *Rhipicephalus*, *Haemaphysalis*, and *Hyalomma* [21, 22]. Studies have reported that rickettsial endosymbionts play an important role in the physiological fitness, population dynamics, and vector competence of their tick hosts [13, 17, 23–25]. In addition, interaction between a rickettsial endosymbiont and another *Rickettsia* influences the transmission of pathogens and the distribution of the endosymbionts [26–28]. While these studies are mostly focused on hard ticks, fewer studies have focused on addressing the presence or interactions of rickettsial bacteria with soft ticks.

*Occidentia* is a new genus within the family Rickettsiaceae, along with *Rickettsia* and *Orientia* [29]. Currently, the only species in the genus *Occidentia* is *Occidentia massiliensis*, which was isolated and characterized from a soft tick *Ornithodoros sonrai*, collected in Senegal [29]. *Occidentia massiliensis* is a gram-negative, obligate intracellular, rod-shaped bacillus with a genome size of 1,469,252 bp [29]. This bacterium has no plasmids but has one chromosome [29]. In addition to their presence in *O*. *sonrai*, *Oc*. *massiliensis* was identified in another soft tick, *Argas japonicus*, collected in Japan [30]. In another recent study, *Oc*. *massiliensis* was identified for the first time in hard ticks, *Africaniella transversale* [31]. These studies suggest that *Oc*. *massiliensis* could be a common endosymbiont in both hard and soft ticks. In this study, we determined whether we could detect *Occidentia* species in *O*. *turicata americanus*. Our study indicates that *Occidentia*-like species was evident in different development stages of *O*. *turicata americanus*, indicating vertical transmission, and in different tick tissues. In addition, we noted that blood feeding induces *Occidentia*-like species 16S rRNA expression in these ticks.

## Results

### PCR amplification with *Rickettsia* 16S rRNA oligonucleotides showed presence of *Occidentia*-like species in *O*. *turicata americanus* ticks

**Fig 1:**
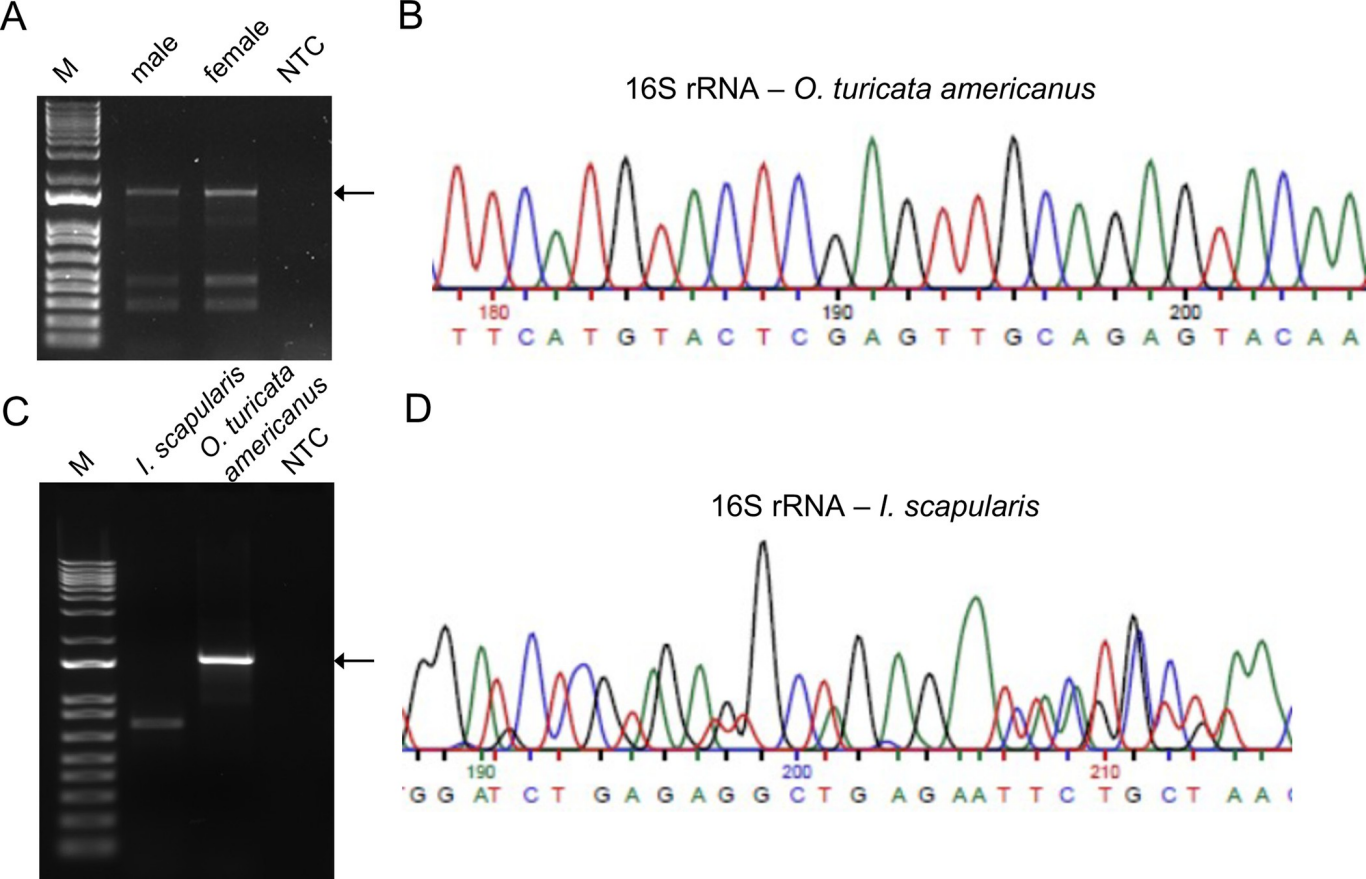
Identification of *Occidentia*-like species in *O*. *turicata americanus* ticks. Agarose gel image showing PCR amplification of 16S rRNA gene fragment from total DNA isolated from unfed *O*. *turicata americanus* adult male or female (A) and unfed *O*. *turicata americanus* adult female or unfed *I*. *scapularis* female tick (C). M indicates DNA marker. NTC indicates no-template control. Arrow in A and C indicates 16S rRNA product in *O*. *turicata americanus* ticks. Sequencing analysis of partial *Occidentia*-like species 16S rRNA sequence from unfed *O*. *turicata americanus* female (B) or *I*. *scapularis* female (D) tick is shown.

Rickettsial endosymbionts have been detected in a wide variety of ticks [16, 31–34]. To address whether *O*. *turicata americanus* ticks harbor any *Rickettsia*, total DNA was isolated from uninfected unfed adult *O*. *turicata americanus* ticks. This DNA was used as a template for PCR amplification with published [35] *Rickettsia* 16S rRNA oligonucleotides (Fig 1). A band at approximately 1500 bp was evident in the DNA samples generated from both male and female *O*. *turicata americanus* ticks (Fig 1A and 1C). Two other prominent PCR amplified bands between 300–500 bp were also evident in the DNA samples generated from both male and female *O*. *turicata americanus* ticks (Fig 1A). All three bands from female ticks were sequenced from both ends. Sequencing of the 1500 bp band gave a 98% match to *O*. *massiliensis* 16S rRNA (Fig 1B, S1A and S2A Figs). We designated this bacterium as *Occidentia*-like species due to its high identity with *Oc*. *massiliensis*. The sequencing results of the lower bands (1000–500 bp) showed weak sequencing peaks. The BLAST searches revealed low sequence coverage and showed non-*Rickettsia*-specific matches (eg. Human transcription factor SREBF1). The 16S rRNA partial sequence from *Occidentia*-like species found in *O*. *turicata americanus* ticks is shown in S3 Fig and submitted to GenBank (accession no. OP799373). Furthermore, to address whether *Ixodes scapularis* ticks also harbor *Occidentia*-like species, total DNA was isolated from uninfected unfed female adult ticks. PCR amplification with 16S rRNA resulted in a band around 750 bp (Fig 1C). Sequencing of this band revealed mixed sequences (Fig 1D and S2B Fig) that showed 83–84% identity with several *Rickettsia* species such as *R*. *tamurae* subp. *buchneri* strain ISO7, *R*. *monacensis* strain Bel-4113 and 77% identity with *O*. *massiliensis*. Overall, these results suggest that *Occidentia*-like species are present in *O*. *turicata americanus*.

### PCR amplification with *Oc*. *massiliensis-*specific *groEL* oligonucleotides confirmed presence of *Occidentia-*like species in *O*. *turicata americanus* ticks

**Fig 2:**
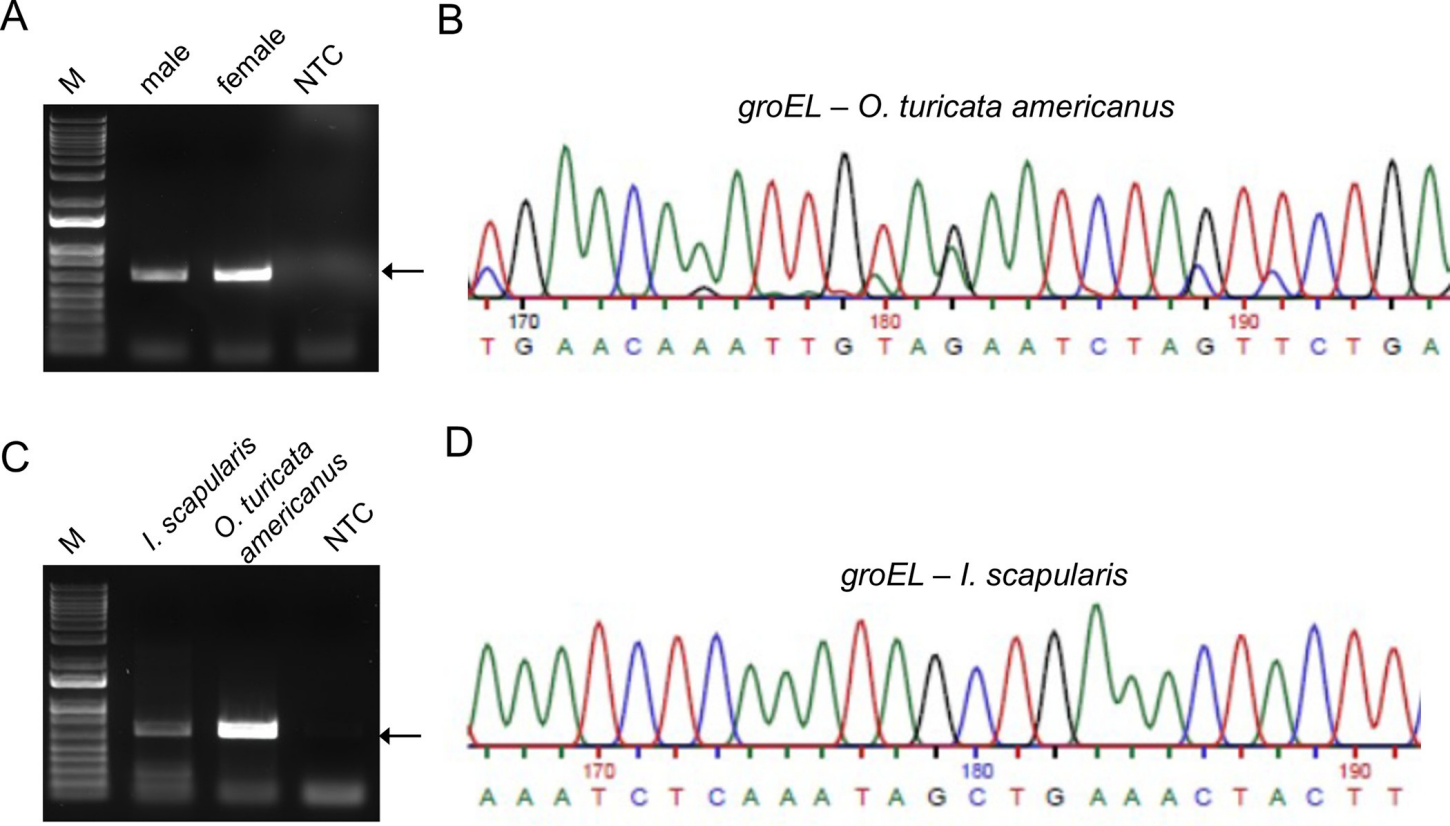
PCR analysis with *Occidentia massiliensis* specific primers confirmed presence of *Occidentia*-like species in *O*. *turicata americanus* ticks. Agarose gel image showing PCR amplification of *groEL* gene fragment from total DNA isolated from unfed *O*. *turicata americanus* adult male and female (A) and unfed *O*. *turicata americanus* female or *I*. *scapularis* female tick (C). M indicates DNA marker. NTC indicates no-template control. Arrow in A and C indicates *groEL* PCR product. Sequencing analysis of partial *Occidentia*-like species *groEL* sequence from unfed *O*. *turicata americanus* female (B) or *I*. *scapularis* female (D) is shown.

To further confirm if *Occidentia*-like species are present in *O*. *turicata americanus* ticks, we performed PCRs with *Oc*. *massiliensis groEL* specific primers [31]. PCR analysis showed an intense band around 650bp in DNA samples generated from both male and female *O*. *turicata americanus* ticks (Fig 2A). Sequencing analysis revealed a clear sequence with 92% match to *Oc*. *massiliensis groEL* gene sequence (Fig 2B and S1B and S4A Figs). The partial sequence of *Occidentia*-like species *groEL* was submitted to GenBank (accession no. OP802357). PCR analysis with *Oc*. *massiliensis* specific primers and DNA sample from *I*. *scapularis* female ticks showed a faint band around 650bp (Fig 2C). Sequencing of this PCR product revealed a sequence with 98–99% identity with the *groEL* gene of several *Rickettsia* species (S4B and S5 Figs). The *groEL* partial sequence for *Occidentia*-like species found in *O*. *turicata americanus* ticks is shown in S6 Fig. Collectively, these results indicate that *Occidentia*-like species are present as endosymbionts in *O*. *turicata americanus* ticks.

### Comparison of *Occidentia-like* species 16S rRNA and *groEL* sequences with *Oc*. *massiliensis* and other species in the Rickettsiaceae family

**Fig 3:**
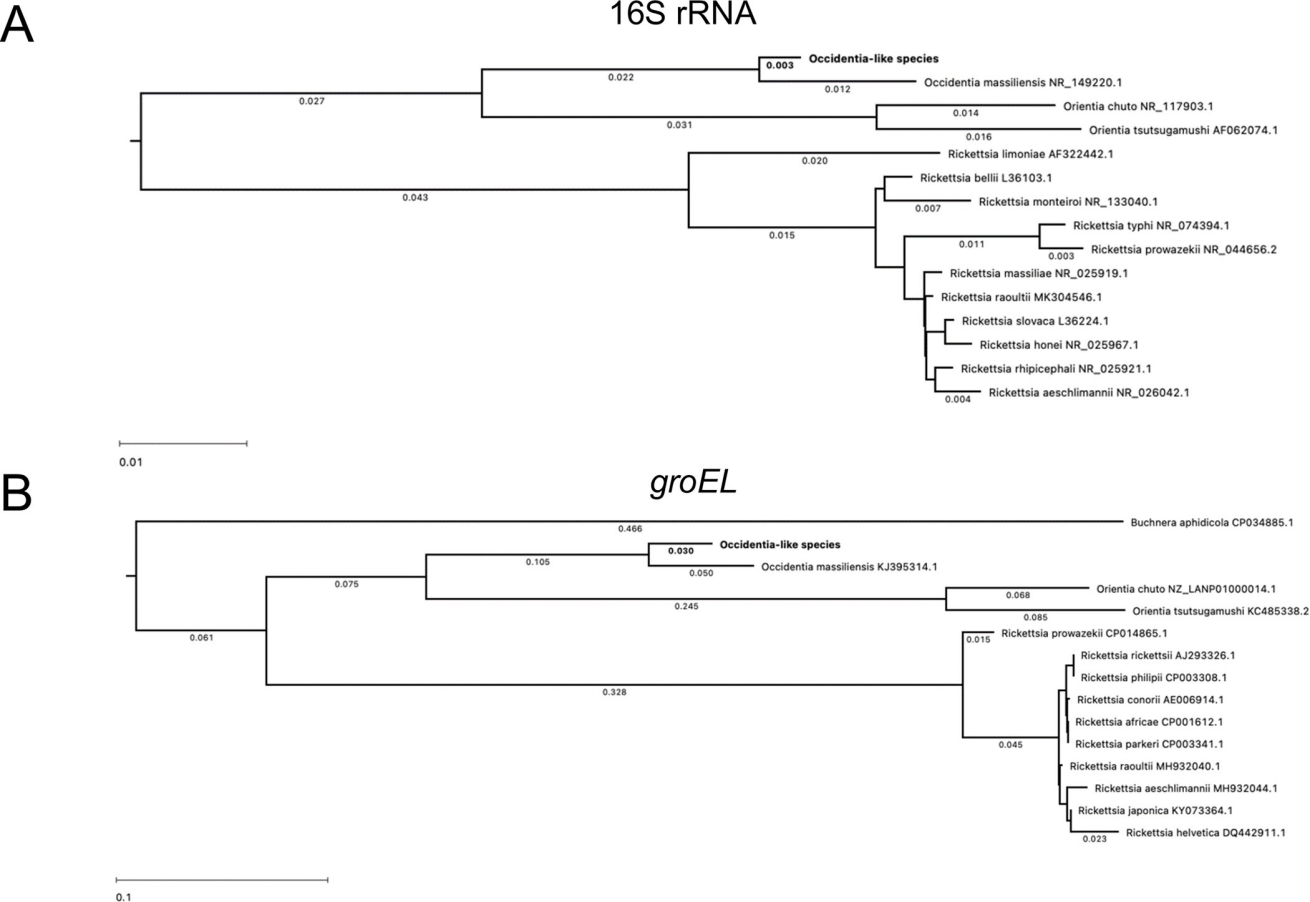
Phylogenetic analysis of *Occidentia*-like species 16S rRNA gene and *groEL* genes nucleotide sequences with other rickettsial bacteria. Phylogenetic trees showing relatedness of *Occidentia*-like species 16S rRNA (A) and *groEL* (B) with *Occidentia massiliensis*, *Orientia chuto*, *Orientia tsutsugamushi*, *Buchnera aphidicola* and other *Rickettsia* spp. is shown. MAFFT (v6.240) was used as the method for multiple sequence alignment and RAxML (v8.2.12) was used to construct the phylogenetic tree. GenBank accession numbers are provided along with the species names.

Fourteen rickettsial species were selected for the comparison of the nucleotide sequence for 16S rRNA gene, which have the highest identity to *Occidentia*-like species. Similarly, 13 rickettsial species and *Buchnera aphidocola* were selected for *groEL* gene, as the *groEL* gene of *Buchnera* has unusually high identity to that of *Occidentia*-like species. All nucleotide sequences were downloaded from GenBank [National Center for Biotechnology Information (NCBI)] and processed for alignment and phylogenetic analysis using DNASTAR software with ClustalW method. We noted that the sequence of 16S rRNA of *Occidentia*-like species is highly conserved, sharing 98.8% identity with *Oc*. *massiliensis* and above 91% identity with *Orientia chuto*, *O*. *tsutsugamushi* and 11 other *Rickettsia* spp. (S1A Fig). The phylogenetic analysis showed that the 16S rRNA sequence from *Occidentia*-like species shares a same clade with *Oc*. *massiliensis* and these two organisms were close to the clade shared by *Orientia chuto* and *O*. *tsutsugamushi* (Fig 3A). The other analyzed rickettsial species formed a different clade (Fig 3A). The *Occidentia*-like species *groEL* sequence was 92.7% identical to that of *Oc*. *massiliensis* (S1B Fig) and between 67.8% to 74% identity with *Orientia chuto*, *O*. *tsutsugamushi*, *Buchnera aphidicola*, and ten other similar *Rickettsia* spp. (S1B Fig). The phylogenetic analysis of *groEL* sequence from *Occidentia*-like species revealed that this bacterium falls within the same clade as *Oc*. *massiliensis*, and these two bacteria seem to form a clade close to *Orientia chuto* and *O*. *tsutsugamushi*, divergent from a clade formed by ten *Rickettsia* spp. (Fig 3B). *Buchnera aphidicola* forms a totally divergent clade from all other analyzed species (Fig 3B).

### Detection of *Occidentia*-like species in different developmental stages of *O*. *turicata americanus* ticks

**Fig 4:**
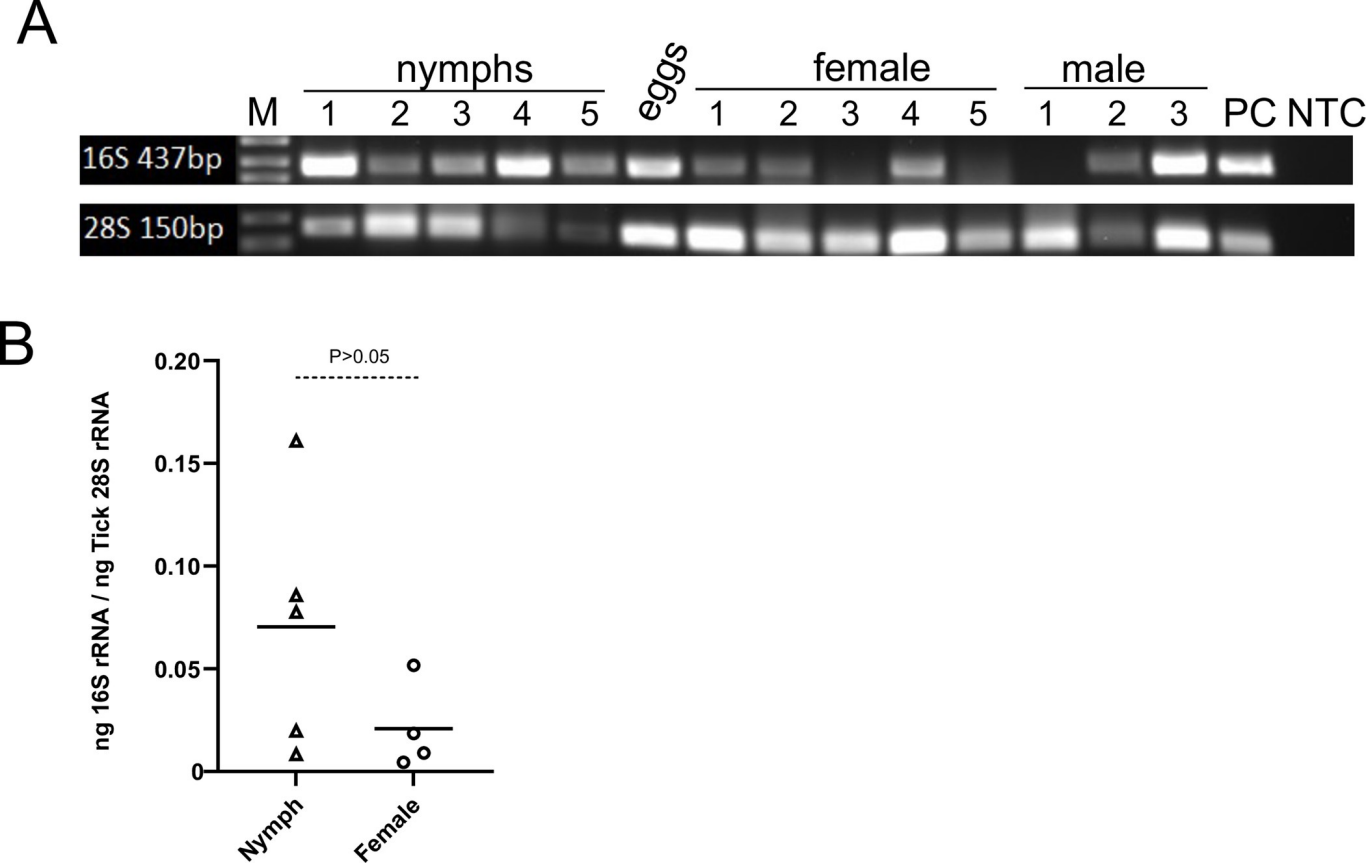
Detection of *Occidentia*-like species in *O*. *turicata americanus* developmental stages. A) Agarose gel image showing PCR amplification of 16S rRNA gene fragment from total DNA isolated from individual (indicated with numbers) unfed *O*. *turicata americanus* nymphs or adult male and female ticks. Eight eggs were pooled and processed together as one sample for total DNA extraction followed by PCR. M indicates DNA marker; PC indicates positive control and NTC indicates no-template control. Full gel image is shown in S7 Fig. Amplification of *O*. *turicata americanus* 28S rRNA serves as a template control. B) QRT-PCR analysis showing levels of *Occidentia*-like species in *O*. *turicata americanus* nymphs and adult females. *Occidentia*-like species 16S rRNA levels were normalized to *O*. *turicata americanus* 28S rRNA levels. Each triangle or circle represents data from sample generated from one tick. Statistical analysis was performed with Student’s t test and P value less than 0.05 was considered as significant.

We then analyzed if *Occidentia*-like species is present in different developmental stages of *O*. *turicata americanus* ticks. Total DNA was isolated from five individual nymphal ticks, five adult female ticks, three adult male ticks, and pool of eight eggs laid by one tick. PCR analysis revealed the presence of *Occidentia-*like species in all five nymphal ticks, four out of five female ticks, two out of three male ticks, and the eggs (Fig 4A and S7A Fig). In addition, sequencing of the representative PCR products confirmed the presence of *Occidentia*-like species in these *O*. *turicata americanus* developmental stages (S7B Fig). Furthermore, quantitative-real time PCR (QRT-PCR) analysis showed no significant differences (P>0.05) in the levels of *Occidentia*-like species between nymphs and female ticks (Fig 4B). These results indicate that *Occidentia*-like species is present in all developmental stages of *O*. *turicata americanus* ticks.

### 16S rRNA transcripts of *Occidentia*-like species are abundantly expressed in tick ovaries than in gut tissue of *O*. *turicata americanus* ticks

**Fig 5:**
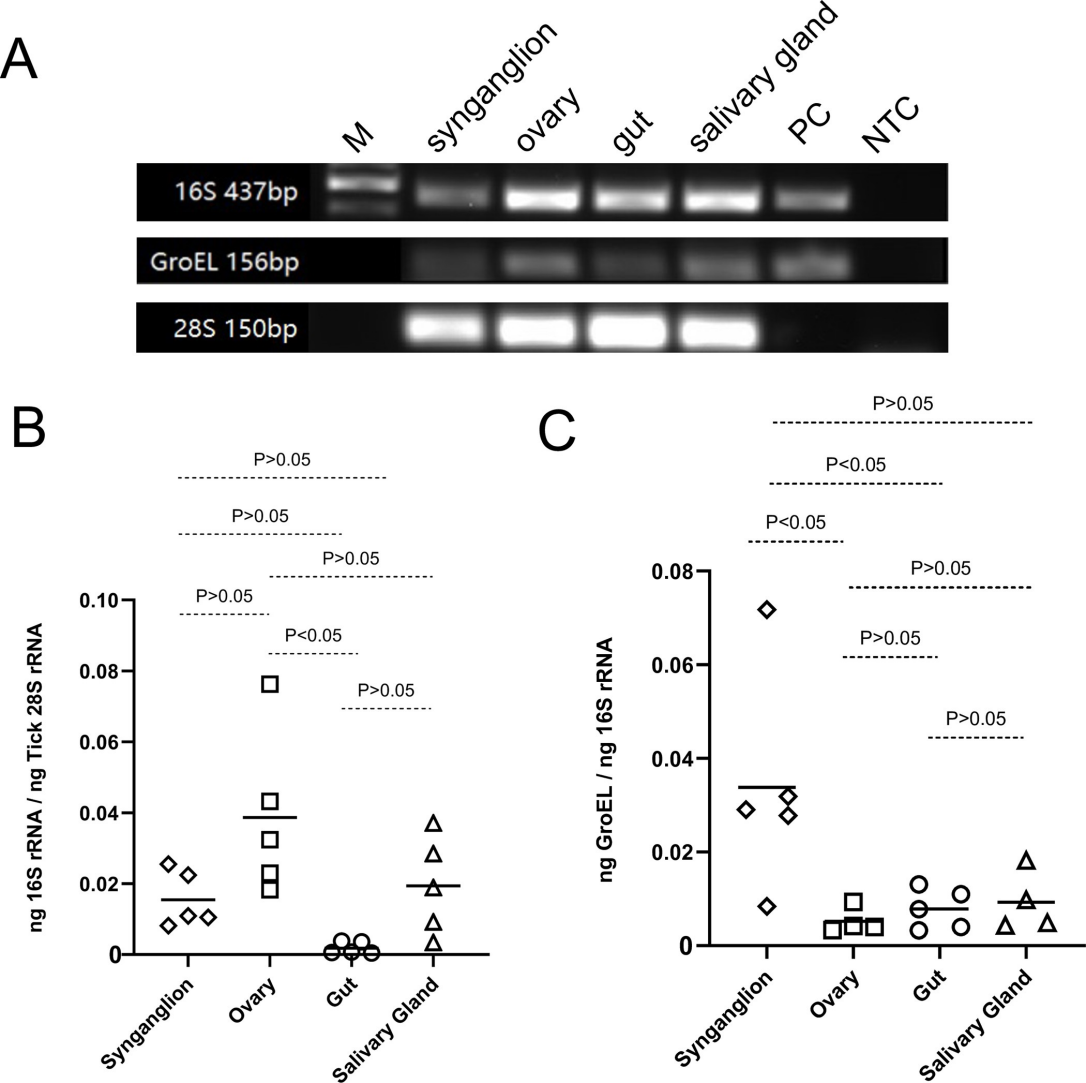
Detection of *Occidentia*-like species transcripts in different *O*. *turicata americanus* tissues. Agarose gel image showing PCR amplification of 16S rRNA gene fragment from total RNA isolated from synganglion, ovaries, salivary glands, and guts from individual fed *O*. *turicata americanus* female ticks. PC indicates positive control and NTC indicates no-template control. Full gel image is shown in S8 Fig. Amplification of *O*. *turicata americanus* 28S rRNA serves as a template control. No positive control was included in 28S rRNA PCR analysis. QRT-PCR analysis with RNA showing *Occidentia*-like species 16S rRNA (B) and *groEL* (C) transcripts in different *O*. *turicata americanus* tissues. *Occidentia*-like species 16S rRNA levels were normalized to *O*. *turicata americanus* 28S rRNA levels. Levels of *Occidentia*-like species *groEL* transcripts were normalized to levels of *Occidentia*-like species 16S rRNA. Each diamond/square/circle/triangle represents data from one individual adult tick tissue. Statistical analysis was performed with Student’s t test and P value less than 0.05 was considered as significant.

To analyze whether transcripts of *Occidentia*-like species are detected in various tick tissues, total RNA from synganglion, ovaries, guts, or salivary glands from five individual fed female ticks was isolated. The RT-PCR with 16S rRNA and *groEL* primers followed by agarose gel electrophoresis confirmed the presence of 16S rRNA and *groEL* transcripts of *Occidentia*-like species in synganglion, ovaries, gut, and salivary gland tissues (Fig 5A and S8 Fig). Sequencing of representative PCR products confirmed presence of transcripts of *Occidentia*-like species in all tested organs (S9 and S10 Figs). RT-PCR results showed variable levels of 16S rRNA and *groEL* transcripts of *Occidentia*-like species in the four tested tissues (Fig 5A). We noted that QRT-PCR showed significantly (P<0.05) increased *Occidentia*-like species 16S rRNA transcripts in ovaries when compared to the levels noted in guts (Fig 5B). However, no significant differences in the *Occidentia*-like species 16S rRNA transcripts were evident between other tested tissue samples (Fig 5B). Furthermore, QRT-PCR showed significantly (P<0.05) increased expression of *Occidentia*-like species *groEL* in *O*. *turicata americanus* synganglion compared to the levels noted in ovaries or gut tissues (Fig 5C). However, no significant difference in the expression of *Occidentia*-like species *groEL* was observed between synganglion or salivary glands (Fig 5C). In addition, no significant difference in the *Occidentia*-like species *groEL* expression was noted between guts or salivary glands (Fig 5B). These results indicate that 16S rRNA transcripts of *Occidentia*-like species are abundantly expressed in *O*. *turicata americanus* ovaries.

### Blood feeding induces 16S rRNA transcripts of *Occidentia*-like species in *O*. *turicata* ticks

**Fig 6:**
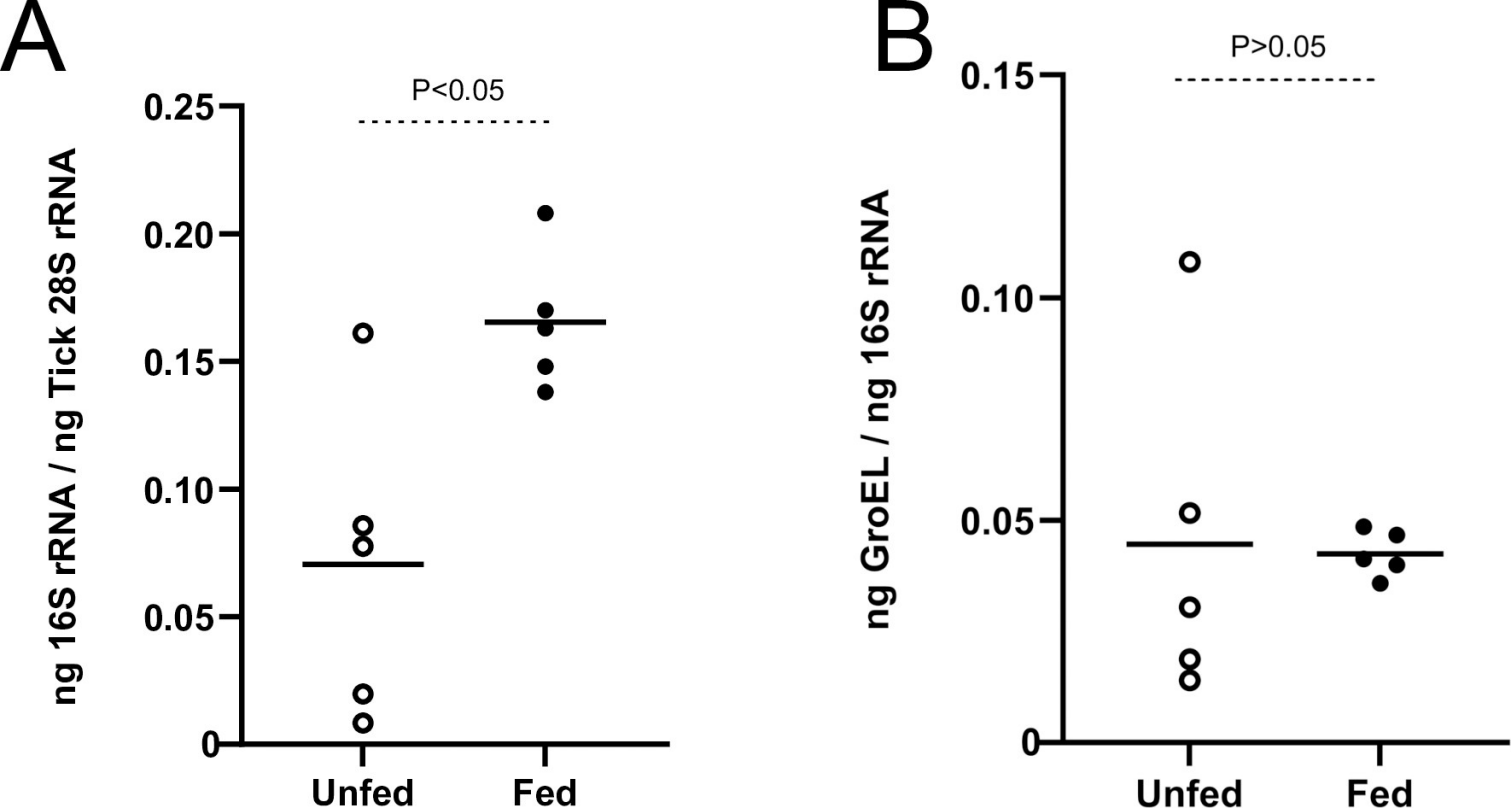
Blood feeding induces *Occidentia-*like species 16S rRNA levels in *O*. *turicata americanus* ticks. QRT-PCR analysis with RNA showing levels of *Occidentia*-like species 16S rRNA (A) and *groEL* (B) transcript levels in unfed and fed *O*. *turicata americanus* nymphal ticks. *Occidentia*-like species 16S rRNA levels were normalized to *O*. *turicata americanus* 28S rRNA levels. Levels of *Occidentia*-like species *groEL* transcripts were normalized to levels of *Occidentia*-like species 16S rRNA. Each open and closed circle represents data from one individual tick. Statistical analysis was performed with Student’s t test and P value less than 0.05 was considered as significant.

To address whether blood feeding has any impact on *Occidentia*-like species transcription, 5 nymphal soft ticks were fed on tick-naïve mice. The repleted ticks were processed for RNA, and cDNA synthesis. QRT-PCR analysis showed a significant (P<0.05) increase in *Occidentia-*like species 16S rRNA transcripts in fed *O*. *turicata americanus* ticks when compared to the levels noted in unfed controls (Fig 6A). Furthermore, QRT-PCR analysis revealed no significant difference in the *Occidentia-*like species *groEL* expression between unfed and fed *O*. *turicata americanus* ticks (Fig 6B). These results suggest that blood meal induces *Occidentia*-like species 16S rRNA expression in *O*. *turicata americanus* ticks.

## Discussion

Rickettsial bacteria are the most common endosymbionts in various tick species [13, 18, 22, 25, 33, 34]. These bacteria show remarkable diversity in different hosts [36]. Some rickettsial bacteria are transovarially transmitted in ticks [13, 18, 22, 25, 33, 34]. Ticks are suggested to be a possible ancestral host for these bacteria [37]. In this study, we report the identification of a rickettsial endosymbiont *Occidentia-*like species in all developmental stages of *O*. *turicata americanus* ticks, including eggs, suggesting a possible transovarially transmitted bacteria in these soft ticks. We noted variable presence of *Occidentia*-like species were noted in adult stages.

Ticks feed on various vertebrate hosts including but not limited to cattle, deer, tortoise, squirrels, snakes, pigs, and prairie dogs [7–10, 38]. The origin of *Occidentia*-like species identified in *O*. *turicata americanus* ticks is currently not known. The identification of *Oc*. *massiliensis* in *O*. *sonrai* ticks collected from rodent burrows [29], in *Africaniella transversale* collected from *Python regius* [31], in *Argas japonicus* collected from nest of Red-rumped swallow (*Hirundo daurica*) [30] and *O*. *turicata americanus* ticks originally collected from burrows of Gopher tortoise used in this current study suggests a complex evolutionary origin of this endosymbiont in different ticks. Currently, the presence of *Occidentia* species in other vertebrate animals or in birds has not been reported. Therefore, the hypothesis on the co-evolution of some ticks with *Occidentia* species, perhaps as an endosymbiont, cannot be ruled out.

The use of two genetic markers (16S rRNA and *groEL*) confirmed the presence of *Occidentia*-like species in *O*. *turicata americanus* ticks. In particular, the oligonucleotides used for *groEL* amplification was specifically designed for the *Oc*. *massiliensis* gene as reported in a previous publication [31]. The authors noted that these oligonucleotides may also amplify *Orientia tsutsugamushi groEL* gene [31]. These oligonucleotides were designed based on the alignment of *groEL* sequences [31]. Phylogenetic analysis performed in this study revealed that the *groEL* sequence of *Occidentia-*like species (identified in this study) is closer to *Oc*. *massiliensis* than to the *O*. *tsutsugamushi* sequence. In addition, the use of 16S rRNA oligonucleotides also resulted in clearly different sequencing peaks that suggests the dominance of *Occidentia-*like species in *O*. *turicata americanus* ticks. Therefore, it is reasonable to assume that the authentic *Occidentia*-like species are present in these ticks.

We noted that *Occidentia*-like species is present in different tick tissues including the synganglion. The presence of bacteria in synganglion is not surprising. A recent study showed that *Ehrlichia muris*-like agent was found to be present in several tick tissues including the synganglion [39]. The role of *Occidentia*-like species in *O*. *turicata americanus* tick synganglion is currently not known. The expression of *Occidentia*-like species *groEL* was significantly upregulated in synganglion compared to the expression noted in guts and ovaries. The GroEL performs chaperonin activity including proper folding of the misfolded proteins [40]. Higher levels of *groEL* suggests active *Occidentia*-like species protein turnover in *O*. *turicata americanus* synganglion. We also noted increased *Occidentia*-like species 16S rRNA levels upon *O*. *turicata americanus* feeding. It is reasonable to hypothesize that *Occidentia*-like species in *O*. *turicata americanus* may use certain nutrients from the blood meal or use energy from arthropod mitochondria to replicate and prepare for transstadial/transovarial transmission in these ticks. It has been reported that levels of endosymbiont *Candidatus Midichloria mitochondrii* was induced upon *I*. *ricinus* blood feeding [41]. In addition, a previous report showed that higher number of *Oc*. *massiliensis* was present close to mitochondria in tick cell line [29]. An extensive literature has shown important contributions by similar endosymbionts in diverse blood feeding arthropods, including various tick species [42]. Endosymbionts contribute in arthropod blood feeding, vector fitness and pathogen interactions [42]. Some of the endosymbionts provide nutrients that are missing in the vertebrate host blood and vitamins to their vector host [42]. Endosymbionts such as *Coxiella* are critical for *A*. *americanum* survival and overall vector fitness [42]. In addition, presence of *Coxiella* is reported to impair transmission of *Ehrlichia chaffiensis* from these ticks [42]. Some endosymbionts are also essential for pathogen development in vectors [42]. Currently, it is not known what biological benefits, if any, that the presence of this endosymbiont provides for its tick host.

In summary, our study shows the presence of *Occidentia*-like species in *O*. *turicata americanus* ticks. The observation of *Occidentia*-like species in *O*. *turicata americanus* opens interesting insights to understand interaction between this rickettsial bacterium and its tick host.

## Methods

### Ticks and mice organism information

*Ornithodoros turicata americanus* nymphs, adult female and male ticks, and eggs were collected from continuously maintained colonies at Old Dominion University, Norfolk, VA. The *O*. *turicata americanus* ticks were originally collected from burrows of the gopher tortoise (*Gopherus polyphemus*) in Florida, U.S.A. These ticks were donated by Dr. J.H. Oliver, Jr., The director of the tick lab at Georgia Southern University in Statesboro, GA., to one of the authors (Dr. Daniel Sonenshine). Dr. J.H. Oliver, Jr., identified these ticks as *O*. *turicata*, Florida strain. However, in view of the findings from Mans et al., [43] we accept the suggestion, originally proposed by Beck et al [11] that *O*. *turicata* from Florida should be considered as a subspecies, *O*. *turicata americanus*. Therefore, throughout the manuscript we have referred to this species as *O*. *turicata americanus*. These ticks were housed in the controlled environment chamber (Parameter Generation and Control, Black Mountain, NC) at 23°C with 95% relative humidity and a 14/10 h light/dark photoperiod regiment. All experiments involving animals were performed in strict accordance with the recommendations in the Guide for the Care and Use of Laboratory Animals of the National Institute of Health [44]. The protocol used in this study (permit number: 10–018) was approved by the Old Dominion University Institutional Animal Care and Use Committee (Animal Welfare Assurance Number: A3172-01). Unfed larvae, nymphs or adult ticks were fed on 6–8 weeks old CD1 mice (Charles River Laboratories, USA). Before tick placement, animals were tranquilized with acepromazine to minimize distress and/or discomfort prior to or during tick feeding. Upon feeding, repleted ticks were collected and processed for DNA or RNA extractions.

### PCR and sequencing

*Ornithodoros turicata americanus* DNA or cDNA was used as templates for the amplification of 16S rRNA and *groEL* gene. Following are the published oligonucleotides used for the PCR amplification of 16S rRNA gene (F 5’ TAAGGAGGTAATCCAGCC 3′ and R 5′ CCTGGCTCAGAACGAA 3′) and *groEL* gene (F 5’ AAAAAAGAAATGTTAGAAGATATTGC 3’ and R 5’ GTACGTACWACTTTAGTTGG 3’) [31, 35]. PCR was performed using the following conditions for the amplification of 16S rRNA gene: initial denaturation at 94 degrees for 5 min followed by 30 cycles of steps including 94 degrees for 1 min, 55 degrees for 1 min, and 72 degrees for 2 min. PCR for *groEL* gene was performed according to the following protocol: initial denaturation at 95 degrees for 5 min followed by 40 cycles of steps including 95 degrees for 30 sec, 45 degrees for 30 sec, and 72 degrees for 1 min. PCR products were later run on 1.2% agarose gels and corresponding bands (~1500 bp for 16S rRNA gene and ~650 for *groEL* gene) were purified using Qiagen QIAquick Gel Extraction Kit (Qiagen, Valencia, USA). PCR products were sequenced from both ends at the Simple Seq core facility (Eurofins MWG Operon Inc., Huntsville, USA).

### Agarose gel electrophoresis

Agarose (Sigma-Aldrich, St. Louis, USA) was dissolved in 1X TAE (Tris-acetate-EDTA) buffer to make agarose gels in 10-centimeter-long trays. We used 1.2% and 1.5% agarose gels to separate and purify PCR products and QRT-PCR products, respectively. After loading each well with PCR or QRT-PCR reaction mix, electrophoresis was performed for ~30 minutes under 80 V.

### Quantitative real-time PCR (QRT-PCR) analysis

QRT-PCR analysis was performed as with CFX OPUS instrument (BioRad, USA) with conditions as described in our previous studies [45–47]. Briefly, total DNA from individual nymphal or adult ticks or pool of eight eggs was extracted with the Qiagen DNeasy Blood & Tissue Kit (Qiagen, Valencia, USA) according to the manufacturer’s instruction. The extracted DNA was used as a template for quantifying the 16S rRNA gene of *Occidentia*-like species using oligonucleotides (10 μM/reaction) (5’ GTTAGAAGTGAAATCCCGAA 3’ and 5’ GAACTGAAGAAAAGCGTCTCCGC 3’. To normalize the amount of template, *O*. *turicata americanus* 28S rRNA gene was quantified as an internal control using oligonucleotides 5’ GATTCCCACTGTCCCTATCTACTATCT 3’ and 5’ GCGACCTCCCACTTATGCTACA 3’. Total RNA from synganglion, ovaries, guts, and salivary glands of adult female ticks was generated using the Bio-Rad Aurum Total RNA Mini Kit (Bio-Rad, Hercules, USA) following the manufacturer’s instruction. RNA was converted to cDNA using Bio-Rad iScript cDNA Synthsis Kit (BioRAD, Hercules, USA). The generated cDNA was used as a template for quantifying the 16S rRNA gene transcripts and *Occidentia*-like species *groEL* gene transcripts using the above-mentioned oligonucleotides with the same internal control to normalize the amount of template. The *groEL* transcripts of *Occidentia*-like species were quantified using oligonucleotides 5’ CACGCTGCGCTCAGATTCGTGAA 3’ and 5’ CACGATCTTTACGTTCTTTTTGC 3’. The protocol for the preparation of cDNA includes priming at 25 degrees for 5 min, reverse transcription at 46 degrees for 20 min, inactivation of reverse transcriptase at 95 degrees for 1 min and holding at 4 degrees. The same QRT-PCR protocol was used for both genes: initial denaturation at 95 degrees for 3 min, 40 cycles of steps including 95 degrees for 10 sec, 58 degrees for 10 sec, and 72 degrees for 30 sec, and melting curve step from 65 degrees to 95 degrees with a 0.5-degree increment for every 5 sec. QRT-PCR was performed using iQ-SYBR Green Supermix (Biorad, USA). A standard curve was generated using 10-fold serial dilutions starting from 1 ng to 0.00001 ng of known quantities of 16S rRNA, *groEL* or 28S rRNA fragments.

### Sequence alignment and phylogenetic analysis

GenBank accession numbers for the sequences used for 16S rRNA are: *Occidentia massiliensis* (NR_149220.1), *Orientia chuto* (NR_117903.1), *Orientia tsutsugamushi* (AF062074.1), *Rickettsia limoniae* (AF322442.1), *R*. *bellii* (L36103.1), *R*. *prowazekii* (NR_044656.2), *R*. *typhi* (NR_074394.1), *R*. *massiliae* (NR_025919.1), *R*. *slovaca* (L36224.1), *R*. *rhipicephali* (NR_025921.1), *R*. *monteiroi* (NR_133040.1), *R*. *honei* (NR_025967.1), *R*. *raoultii* (MK304546.1), *R*. *aeschlimannii* (NR_026042.1). GenBank accession numbers for the sequences used for *groEL* are: *Occidentia massiliensis* (KJ395314.1), *Orientia tsutsugamushi* (KC485338.2), *Orientia chuto* (NZ_LANP01000014.1), *Rickettsia prowazekii* (CP014865.1), *R*. *africae* (CP001612.1), *R*. *parkeri* (CP003341.1), *R*. *philipii* (CP003308.1), *R*. *raoultii* (MH932040.1), *R*. *conorii* (AE006914.1), *R*. *aeschlimannii* (MH932044.1), *R*. *japonica* (KY073364.1), *R*. *helvetica* (DQ442911.1), *R*. *rickettsii* (AJ293326.1) and *Buchnera aphidicola* (CP034885.1). These sequences were downloaded from GenBank and processed with MegAlign Pro (v17.3.1) by DNASTAR software for the alignment and for the phylogenetic analysis. MAFFT (v6.240) was used as the method for multi-alignment of the sequences selected, using the sequence of both genes of *Occidentia*-like species as the reference sequence. RAxML (v8.2.12) was used as a maximum likelihood method for the phylogenetic analyses, using 100 iterations for bootstrapping. The substitution model used is GTR plus optimization of substitution rates, plus GAMMA model of rate heterogeneity with estimate of proportion of invariable sites (GTR + GAMMA + I).

### Tick dissection

*Ornithodoros turicata americanus* synganglion, ovaries, gut and salivary gland tissues were dissected from freshly fed individual adult female ticks in sterile 1x phosphate buffer saline. All dissected organs were washed 3 times in sterile 1X PBS and then placed in RNA lysis solution. Soon after dissection, tick salivary glands, synganglion and ovaries were placed in a sterile 1X PBS and washed for an additional three times before placing in RNA lysis solution. Guts were ruptured on a separate slide to release all luminal contents including blood and then washed 3 times in 1x PBS and placed in RNA lysis solution (Bio-Rad Aurum Total RNA Mini Kit, BioRad, USA). All samples were processed for RNA extractions. RNA samples were later converted to cDNA followed by QRT-PCR analysis.

### Statistical analysis

All quantitative data were evaluated for statistical analysis using GraphPad Prism 9 software and Microsoft Excel. To compare two means, a non-paired Student’s t test was performed and P values of <0.05 were considered significant. Wherever necessary, statistical test and P values used are reported.

## Supporting information

S1 Fig Nucleotide sequence divergence and percent identity of *Occidentia*-like species 16S rRNA and *groEL* with other rickettsial bacteria. Percent identity (horizontally above black box) and divergence (vertically below black box) of *Occidentia*-like species 16S rRNA (A) and *groEL* (B) in comparison to other rickettsial bacteria is shown. Percent identity and divergence was generated based on MAFFT (v6.240) multiple sequence alignment. GenBank accession numbers are provided along with the species names.
(TIF)

S2 Fig Sequence analysis of *Occidentia-*like species 16S rRNA sequence from *O*. *turicata americanus* and *I*. *scapularis*. Sequencing chromatograms for *Occidentia*-like species 16S rRNA from *O*. *turicata americanus* (A) and *I*. *scapularis* (B) are shown.
(TIF)

S3 Fig Sequence of *Occidentia*-like species 16S rRNA. The partial nucleotide sequence of *Occidentia*-like species 16S rRNA obtained by sequencing PCR products with total DNA isolated from adult female *O*. *turicata americanus* is shown.
(TIF)

S4 Fig Sequence analysis of *Occidentia-*like species *groEL* from *O*. *turicata americanus* and *I*. *scapularis*. Sequencing chromatograms for *Occidentia*-like species *groEL* from *O*. *turicata americanus* (A) and *I*. *scapularis* (B) is shown.
(TIF)

S5 Fig BLAST search performed with *groEL* sequence form *I*. *scapularis*. BLAST search with *groEL* sequence from *I*. *scapularis* showed sequences related to several *Rickettsia* with 98–92% identity.
(TIF)

S6 Fig Sequence of *Occidentia*-like species *groEL*. The partial nucleotide sequence of *Occidentia*-like species *groEL* obtained by sequencing PCR products with total DNA isolated from adult female *O*. *turicata americanus* is shown.
(TIF)

S7 Fig 16S rRNA sequence from different developmental stages of *O*. *turicata americanus* ticks. Full agarose gel image showing amplification of *Occidentia*-like species 16S rRNA (A) and *O*. *turicata* 28S rRNA (B) in *O*. *turicata americanus* nymphs, eggs, adult male and female is shown. C) Representative sequencing chromatograms are shown. Partial gel image is shown in Fig 4A.
(TIF)

S8 Fig PCR amplification of 16S rRNA from different *O*. *turicata americanus* tissues. Full agarose gel image showing amplification of *Occidentia*-like species 16S rRNA and *groEL* and *O*. *turicata* 28S rRNA in *O*. *turicata americanus* synganglion (SY), ovaries (OV), guts (MG) and salivary glands (SG) is shown. Partial image is shown in Fig 5A.
(TIF)

S9 Fig Sequence analysis of *Occidentia-*like species 16S rRNA sequence from *O*. *turicata americanus* tissues. Sequencing chromatograms for *Occidentia*-like species 16S rRNA in *O*. *turicata americanus* synganglion (SY), ovary (OV), gut (MG) and salivary glands (SG) is shown.
(TIF)

S10 Fig Sequence analysis of *Occidentia-*like species *groEL* sequence from *O*. *turicata americanus* tissues. Sequencing chromatograms for *Occidentia*-like species *groEL* in *O*. *turicata americanus* synganglion (SY), ovary (OV), gut (MG) and salivary glands (SG) is shown.
(TIF)
