# Supplementary figures and images for: Identification of a rickettsial endosymbiont in a soft tick Ornithodoros turicata americanus
Source: PLoS One. 2022 Dec 6;17(12):e0278582. doi: 10.1371/journal.pone.0278582 (PMC9725135; doi:10.1371/journal.pone.0278582)

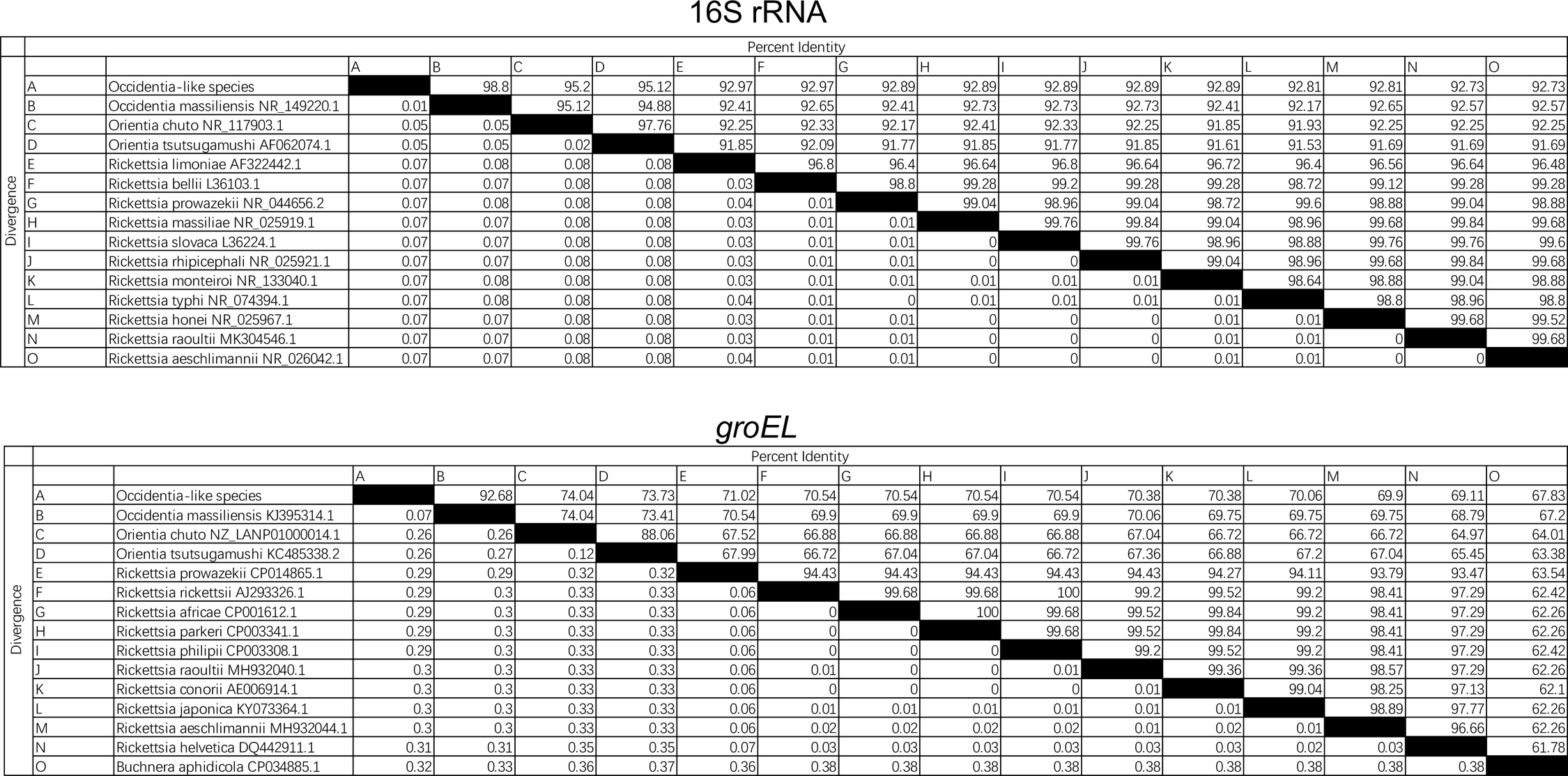

Supplement: S1 Fig — Percent identity (horizontally above black box) and divergence (vertically below black box) of Occidentia-like species 16S rRNA (A) and groEL (B) in comparison to other rickettsial bacteria is shown. Percent identity and divergence was generated based on MAFFT (v6.240) multiple sequence alignment. GenBank accession numbers are provided along with the species names. (TIF) [file pone.0278582.s001.tif]

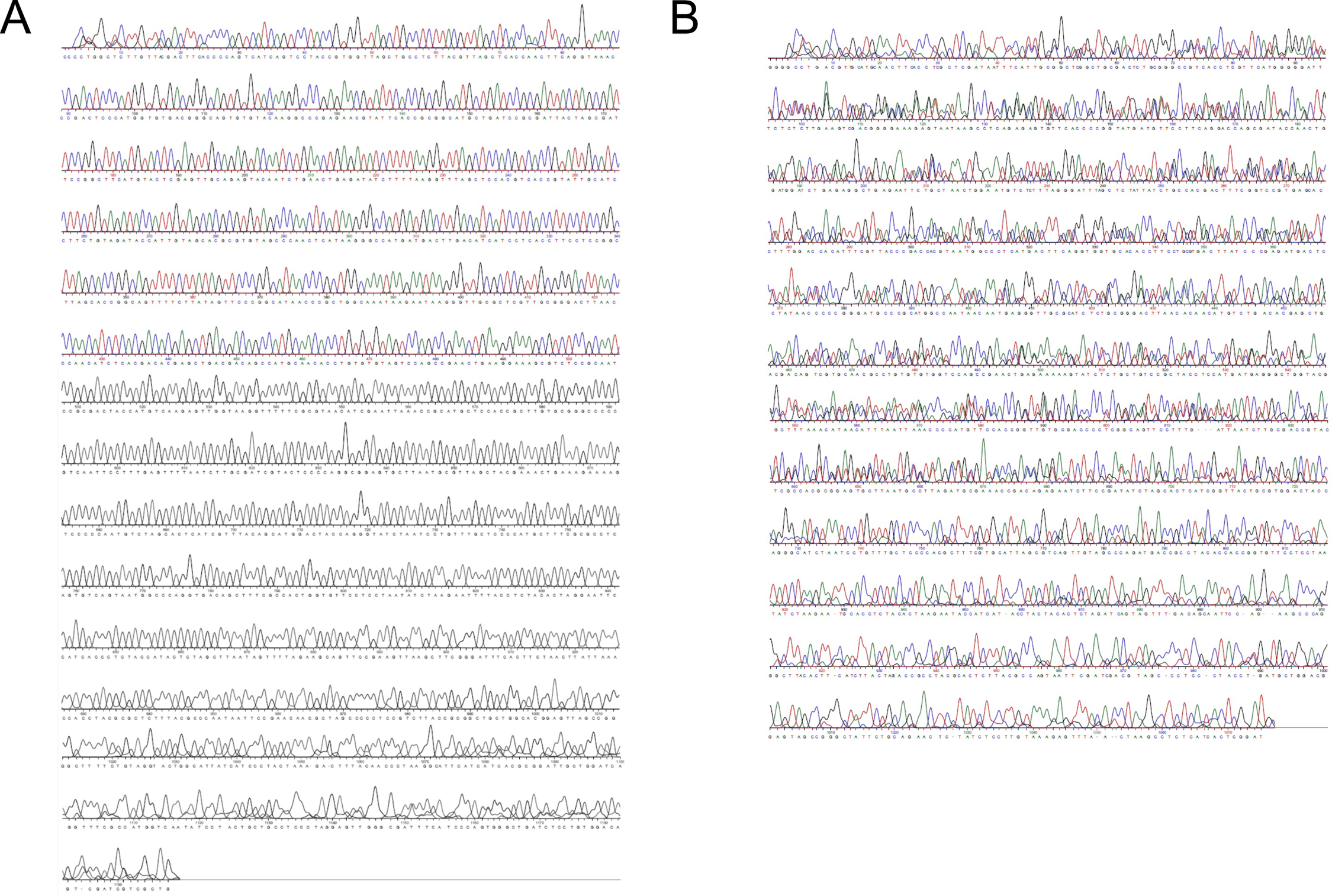

Supplement: S2 Fig — Sequencing chromatograms for Occidentia-like species 16S rRNA from O. turicata americanus (A) and I. scapularis (B) are shown. (TIF) [file pone.0278582.s002.tif]

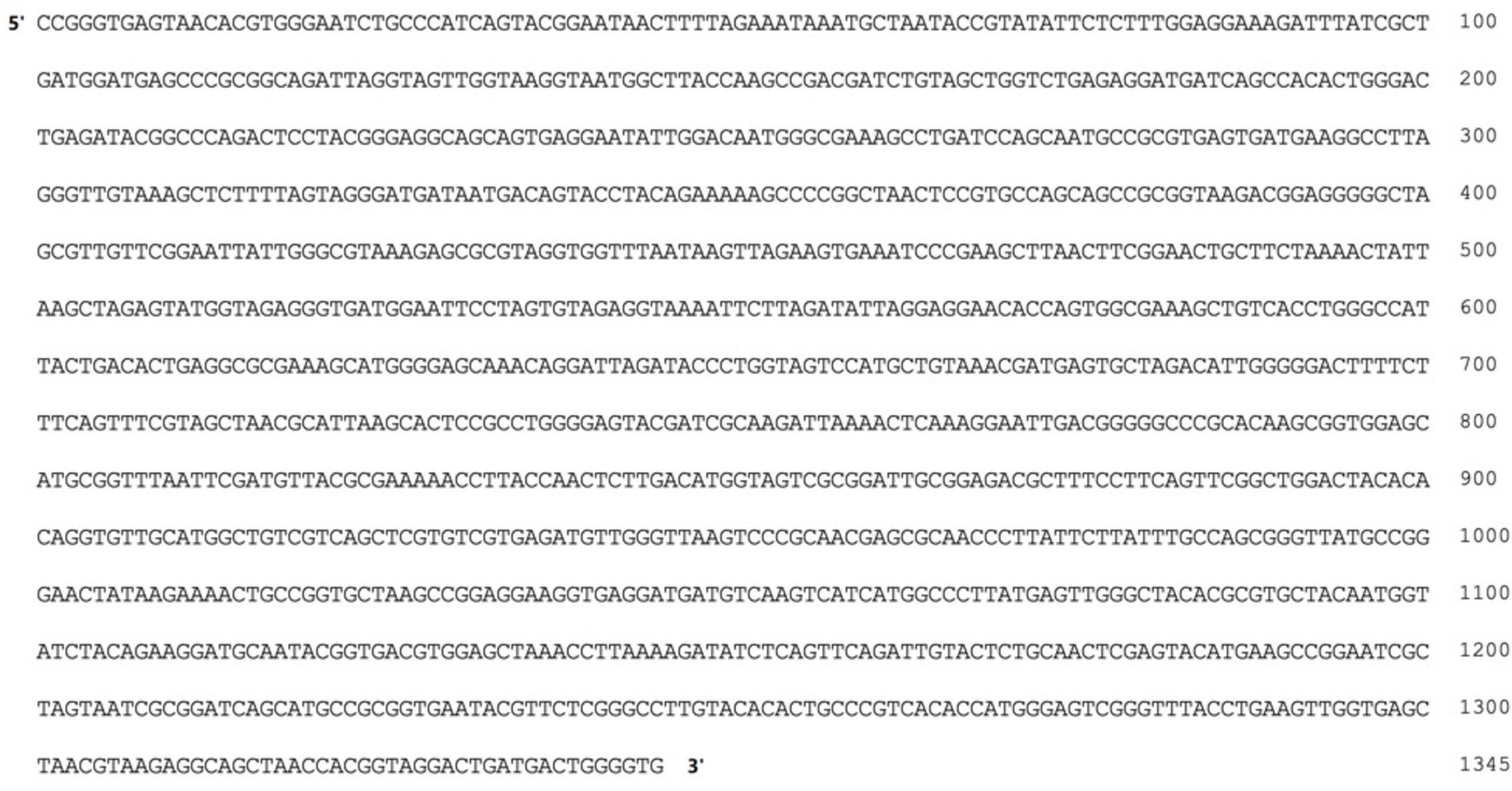

Supplement: S3 Fig — The partial nucleotide sequence of Occidentia-like species 16S rRNA obtained by sequencing PCR products with total DNA isolated from adult female O. turicata americanus is shown. (TIF) [file pone.0278582.s003.tif]

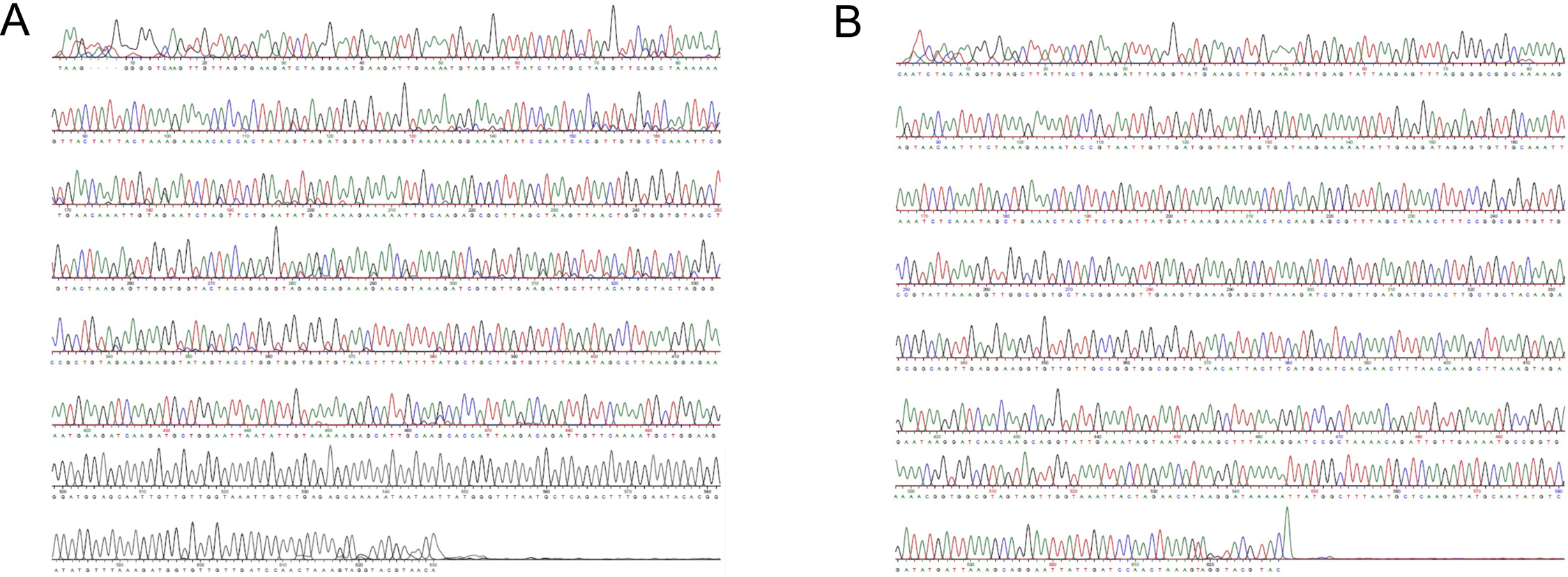

Supplement: S4 Fig — Sequencing chromatograms for Occidentia-like species groEL from O. turicata americanus (A) and I. scapularis (B) is shown. (TIF) [file pone.0278582.s004.tif]

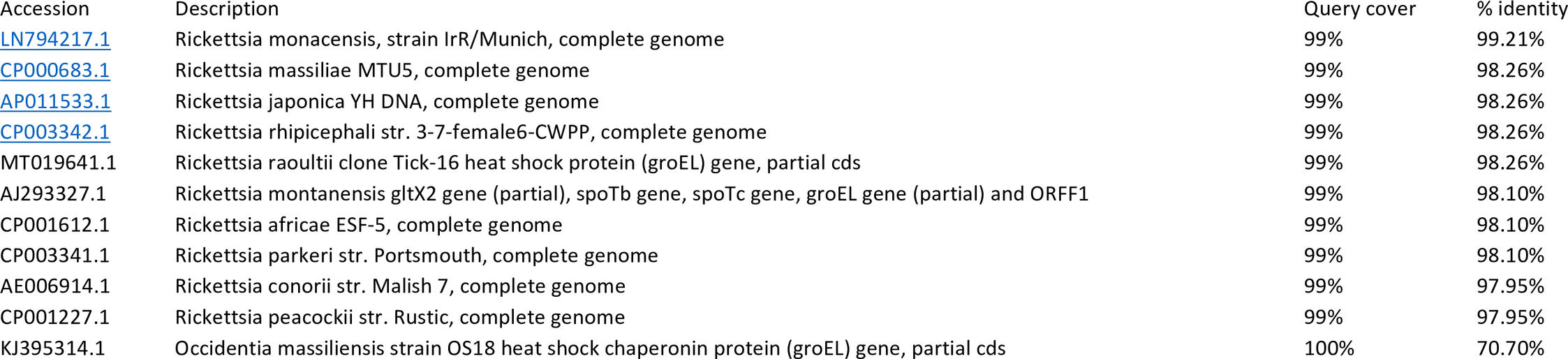

Supplement: S5 Fig — BLAST search with groEL sequence from I. scapularis showed sequences related to several Rickettsia with 98–92% identity. (TIF) [file pone.0278582.s005.tif]

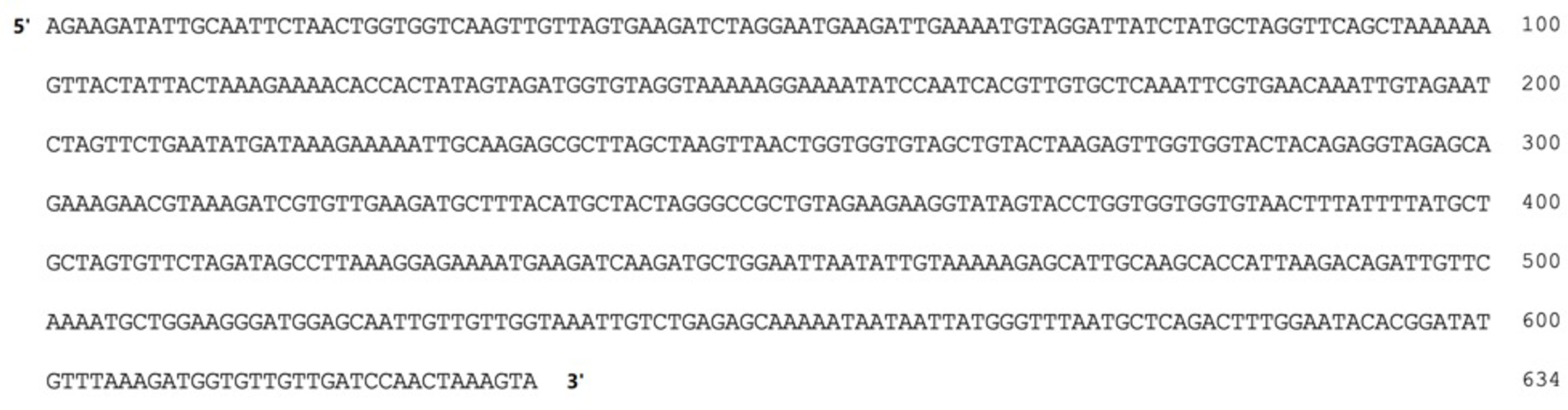

Supplement: S6 Fig — The partial nucleotide sequence of Occidentia-like species groEL obtained by sequencing PCR products with total DNA isolated from adult female O. turicata americanus is shown. (TIF) [file pone.0278582.s006.tif]

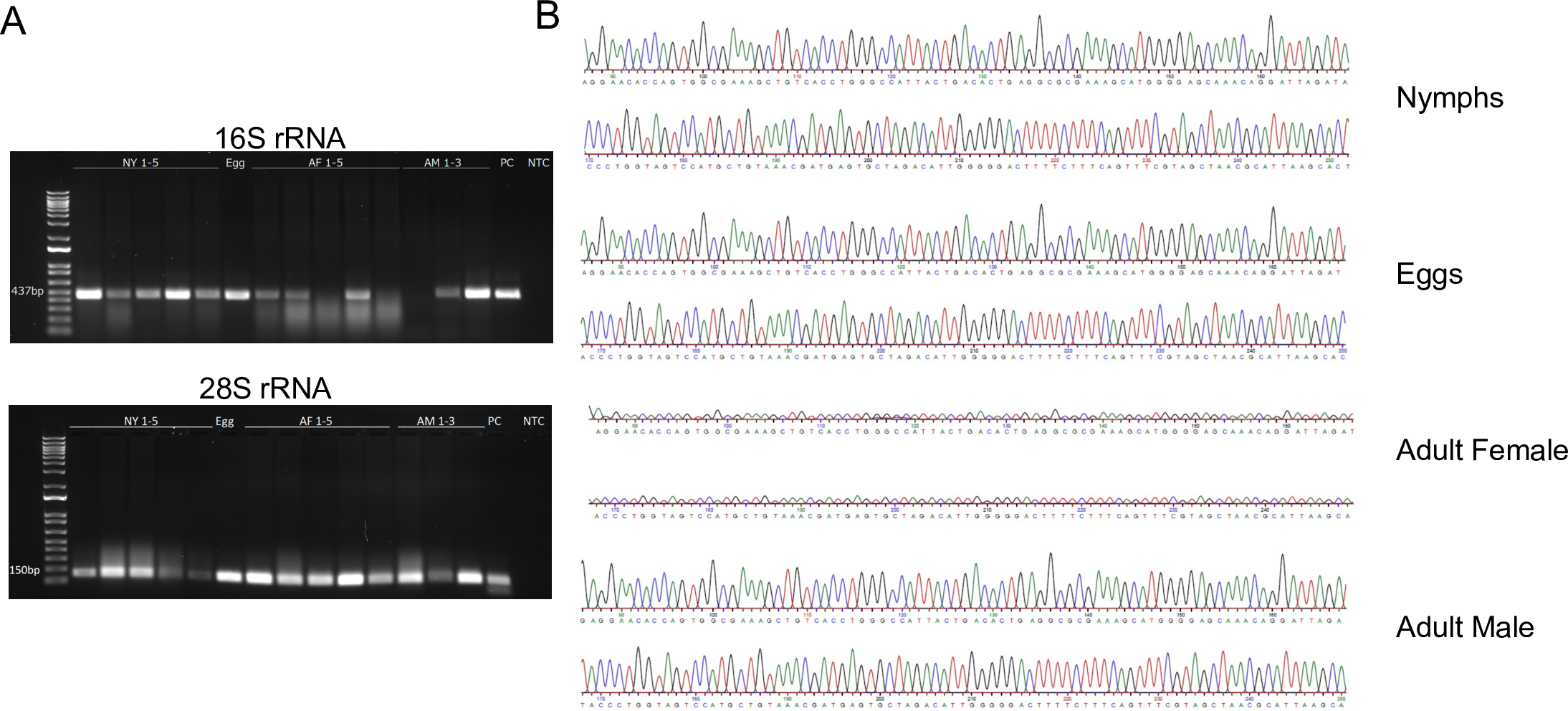

Supplement: S7 Fig — Full agarose gel image showing amplification of Occidentia-like species 16S rRNA (A) and O. turicata 28S rRNA (B) in O. turicata americanus nymphs, eggs, adult male and female is shown. C) Representative sequencing chromatograms are shown. Partial gel image is shown in Fig 4A. (TIF) [file pone.0278582.s007.tif]

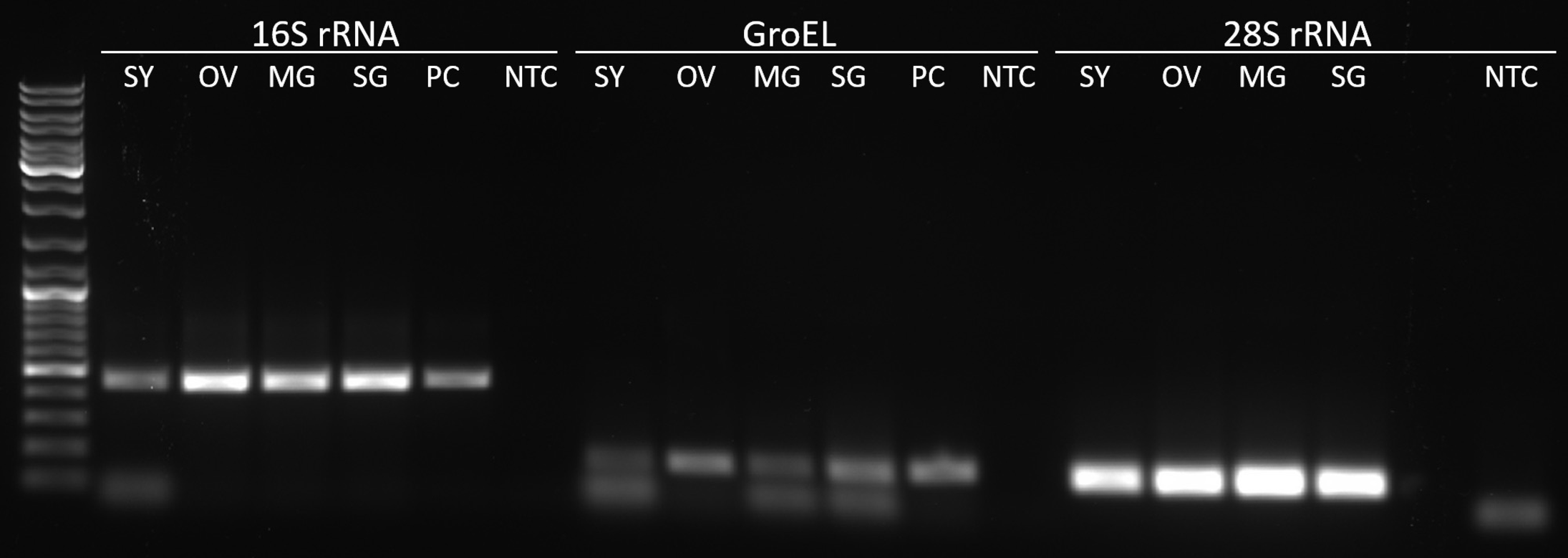

Supplement: S8 Fig — Full agarose gel image showing amplification of Occidentia-like species 16S rRNA and groEL and O. turicata 28S rRNA in O. turicata americanus synganglion (SY), ovaries (OV), guts (MG) and salivary glands (SG) is shown. Partial image is shown in Fig 5A. (TIF) [file pone.0278582.s008.tif]

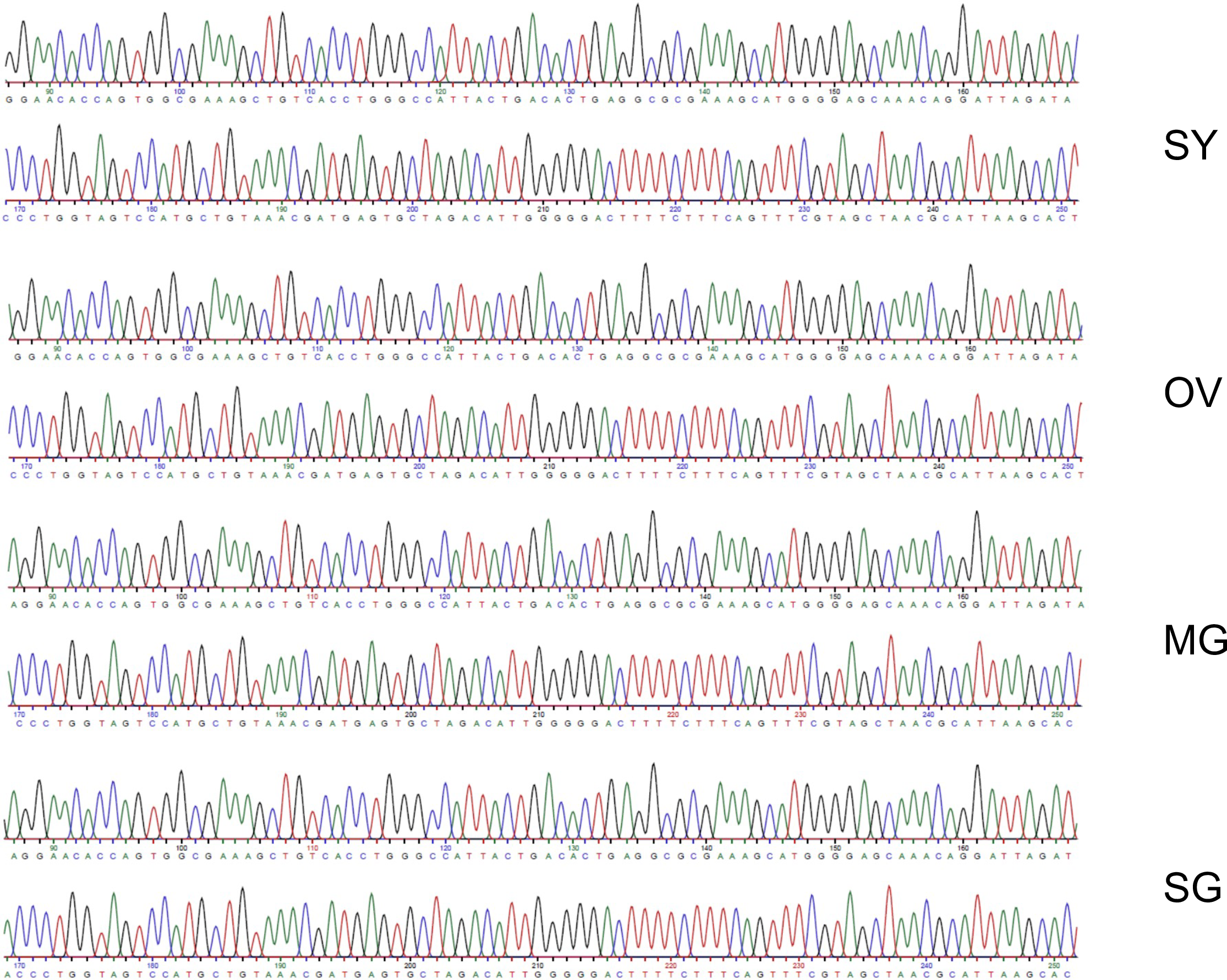

Supplement: S9 Fig — Sequencing chromatograms for Occidentia-like species 16S rRNA in O. turicata americanus synganglion (SY), ovary (OV), gut (MG) and salivary glands (SG) is shown. (TIF) [file pone.0278582.s009.tif]

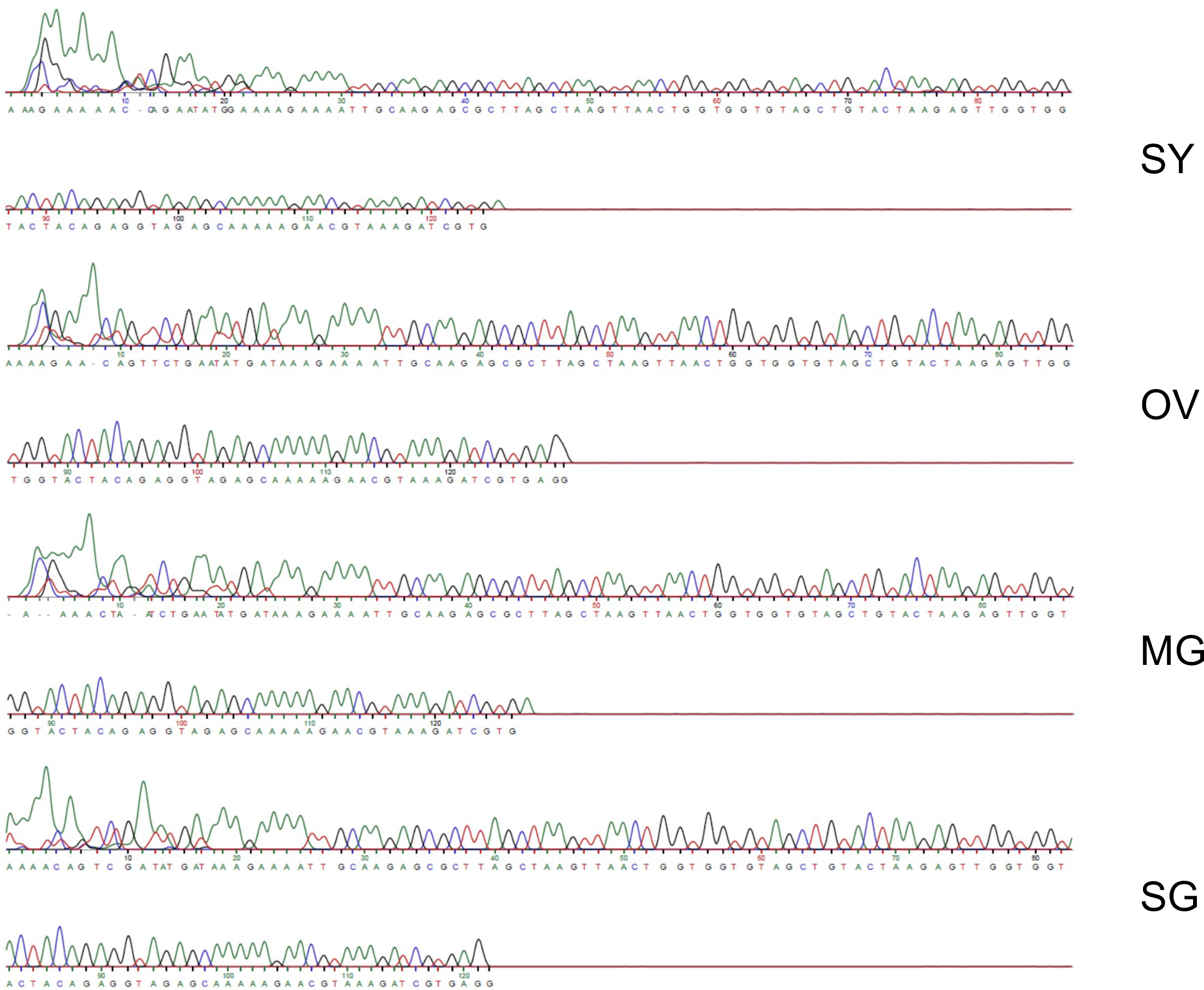

Supplement: S10 Fig — Sequencing chromatograms for Occidentia-like species groEL in O. turicata americanus synganglion (SY), ovary (OV), gut (MG) and salivary glands (SG) is shown. (TIF) [file pone.0278582.s010.tif]
